# Sub-nanometer surface chemistry and orbital hybridization in lanthanum-doped ceria nano-catalysts revealed by 3D electron microscopy

**DOI:** 10.1038/s41598-017-05671-9

**Published:** 2017-07-14

**Authors:** Sean M. Collins, Susana Fernandez-Garcia, José J. Calvino, Paul A. Midgley

**Affiliations:** 1 Department of Materials Science and Metallurgy, University of Cambridge, 27 Charles Babbage Road, Cambridge, CB3 0FS United Kingdom; 2 Departamento de Ciencia de los Materiales, Ingeniería Metalúrgica y Química Inorgánica, Facultad de Ciencias, Universidad de Cadiz, Campus Río San Pedro, Puerto Real (Cádiz) E-11510 Spain

## Abstract

Surface chemical composition, electronic structure, and bonding characteristics determine catalytic activity but are not resolved for individual catalyst particles by conventional spectroscopy. In particular, the nano-scale three-dimensional distribution of aliovalent lanthanide dopants in ceria catalysts and their effect on the surface electronic structure remains unclear. Here, we reveal the surface segregation of dopant cations and oxygen vacancies and observe bonding changes in lanthanum-doped ceria catalyst particle aggregates with sub-nanometer precision using a new model-based spectroscopic tomography approach. These findings refine our understanding of the spatially varying electronic structure and bonding in ceria-based nanoparticle aggregates with aliovalent cation concentrations and identify new strategies for advancing high efficiency doped ceria nano-catalysts.

## Introduction

There is a central tension in chemical microanalysis between comprehensive analysis of sufficient quantities for representative findings and sensitivity to the spatially varying details of the sub-nanometer chemical bonding environment. Yet it is the reactive layer of the first few unit cells of a material’s surface that plays the crucial role in catalyst performance. Often techniques that recover fine nanometer- to atomic-scale structural detail, like electron tomography and atom probe tomography, are limited to nanometer volumes, while techniques that offer sufficient spectral sensitivity for bonding and oxidation state analysis, such as X-ray photoelectron spectroscopy (XPS) and near edge X-ray absorption fine structure (NEXAFS), are limited to ensemble surface measurements or bulk transmission observations. Here, using scanning transmission electron micrscopy (STEM), we combine three-dimensional (3D) structural characterization of entire multi-particle catalyst aggregates with electron energy loss spectroscopy (EELS) analysis of bonding characteristics and oxidation states with sub-nanometer precision. This exploration of composition, bonding, and oxidation state changes addresses significant gaps in the metrology of aliovalent doped ceria nanoparticle catalysts.

Doped ceria materials have been developed extensively for a variety of significant redox processes in catalysis (e.g. automotive three-way catalysts^1^, soot and CO oxidation^2, 3^, and water gas shift reactions^4, 5^) and fuel cells^6^. Ceria-based catalysts often function as both a catalyst support and also play an active role in catalysis where active sites on the surface, particularly involving mobile oxygen species, participate in reactive processes^5, 7^. The incorporation of La or other lanthanide aliovalent dopants shows significant improvement in the catalytic performance of nanoparticle ceria catalysts^8, 9^. However, while the role of La and similar dopants has been studied in a number of ensemble, bulk, and transmission measurements^10–15^, the 3D details of the surface bonding environment in catalyst particle aggregates larger than >20 nm has not been resolved^16, 17^. Particularly, a mounting body of evidence suggests that the surface and bulk compositions and oxidation states of Ce and La cations are not the same in mixed cation solid solutions^11, 18^.

High dopant concentrations have often been explored to demonstrate effects of the dopant in bulk and ensemble measurements^10, 11^. As the need for efficient, economical catalysts increases the demand for materials with low-dopant concentrations, spatially-resolved measurements of the surface chemistry of ceria with low La dopant loadings are instrumental in further developing these important catalysts. Here, we use a new electron tomography approach combining spectroscopic analysis at a single sample orientation with morphological electron tomography to reveal, with sub-nanometer precision, the La cation surface segregation, the Ce^3+^ surface oxidation state, and differences in Ce(*4f*)-O(*2p*) bonding in the surface and bulk of catalyst multi-particle aggregates ca. 100 nm in size containing ca. 10% La^19^.

Efficient use of spectroscopic analysis is particularly important in the electron tomographic analysis of nano-ceria. Pure ceria exhibits a significant electron dose-rate dependent surface reduction reaction^20^. Here, we avoided this characterization challenge by acquiring a single spectrum-image with no repeated exposure at long electron beam dwell times. Moreover, the model-based tomography approach used for data analysis enabled the recovery of high quality spectra from high-noise, but over-sampled, experimental signals. Model-based approaches to tomography have shown significant promise in reducing dose and acquisition time requirements in X-ray energy dispersive spectroscopy^21^. Advancing this approach to EELS of multi-nanoparticle aggregates required further innovation, incorporating non-linear electron scattering effects directly in the electron tomographic reconstruction process.

Building on previous examinations of the distribution of La cations in doped ceria^19^, this analysis of low-concentration La-doped ceria (LDC) nanoparticle aggregates lays out direct experimental evidence for surface enrichment of aliovalent La-dopants, implicating dopant cations specifically in the increased surface reactivity of doped ceria. La cations are present throughout the doped nanoparticles, associated with extrinsic oxygen vacancy formation (one oxygen vacancy for every two La^3+^ dopants). However, additional oxygen vacancies appear at the surface, bearing a close resemblance to oxygen vacancy formation common at the surfaces of pure ceria. We report changes in the localization of Ce(*4f*) orbitals, anticipated from measurements of highly doped ceria, now measured with sub-nanometer precision at the surface in lower La-content phases. Together, these observations suggest new opportunities for promoting surface reactivity with lanthanide dopants with minimal need for bulk or homogeneous doping.

## Results

**Figure 1:**
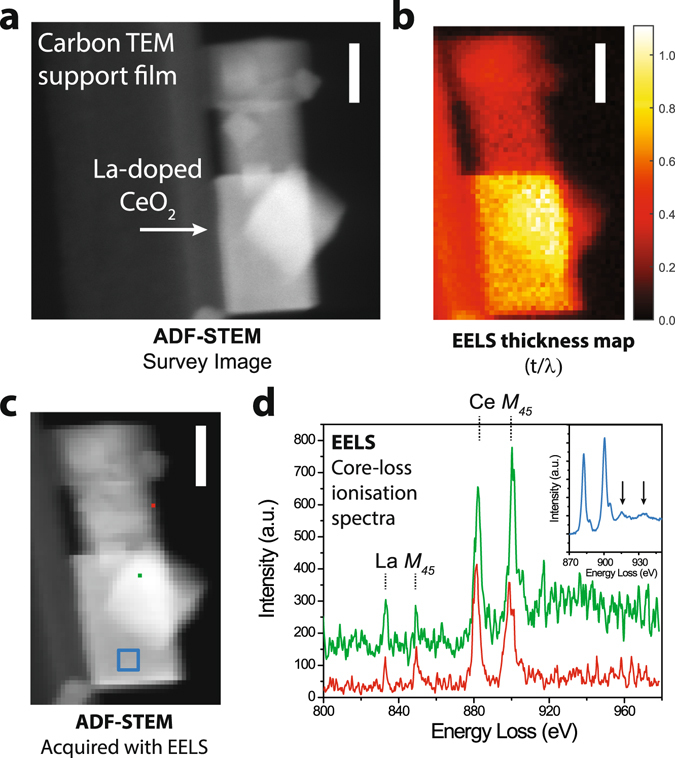
Overview of the La-doped ceria nanoparticle sample and the recorded EELS signals. (**a**) ADF-STEM image of La-doped CeO_2_ nanoparticle aggregate at the edge of a lacey carbon support. (**b**) Thickness map of the aggregate determined by EELS analysis, in units of the inelastic mean free path (*t*/*λ*). (**c**) ADF-STEM image acquired simultaneously with EELS, highlighting the locations of the point spectra and selected area spectra shown in (**d**). (**d**) Point (red, green) and selected area spectra (blue, inset) at the La and Ce *M*_45_ ionisation edges. These spectra were aligned and corrected for artifacts but were not background subtracted. The arrows (inset) emphasize the plural scattering features in the post-edge spectral window. Scale bars are 25 nm.

Figure 1 presents an overview of the characteristics of the LDC particles and the acquired data quality. Figure 1a is an annular dark field (ADF) STEM image of a particle aggregate on the edge of a lacey carbon support film, used to minimize trajectories passing through support material in an effort to eliminate background contributions to the recorded spectra at the O, La, and Ce ionization edges of interest. The aggregates throughout the LDC samples consisted of cubic and rectangular prism shaped particles with side lengths of approximately 25–50 nm. At the electron beam acceleration voltage used (80 kV), the particle size and chemical characteristics determined the ratio of the sample thickness and the inelastic scattering mean free path (*t*/*λ*) to be between typically 0.5 and 1.0 (see Fig. 1b). For conventional EELS analysis, these samples were therefore ‘thick’ and any assumption that multiple inelastic scattering (plural scattering) events were negligible contributions to the recorded spectra is invalid.

Figure 1c,d highlight the acquired data quality of single pixel spectra at the La and Ce *M*_45_ ionization edges. The noise level precluded confident assessment of the EELS near edge fine structure. However, for point spectra taken near the edge of the particle aggregate, the low energy Ce *M*_5_ peak showed higher relative intensity than the *M*_4_ peak at higher energy loss, whereas in the bulk, the peak intensity ratios were reversed. These observations indicated likely Ce^3+^ content at the surface and Ce^4+^ content in the bulk^22^. Integration over a larger area (inset) revealed substantial plural scattering contributions in the post-edge energy window^11^. Plural scattering can be described as a convolution of the inelastic scattering distribution at low energies and the scattering distribution at the core ionization edge^23^, resulting in a shifted and broadened intensity distribution at energies beyond the ionization edge. Due to the noise in the raw spectra, the La content could not be assessed with confidence.

**Figure 2:**
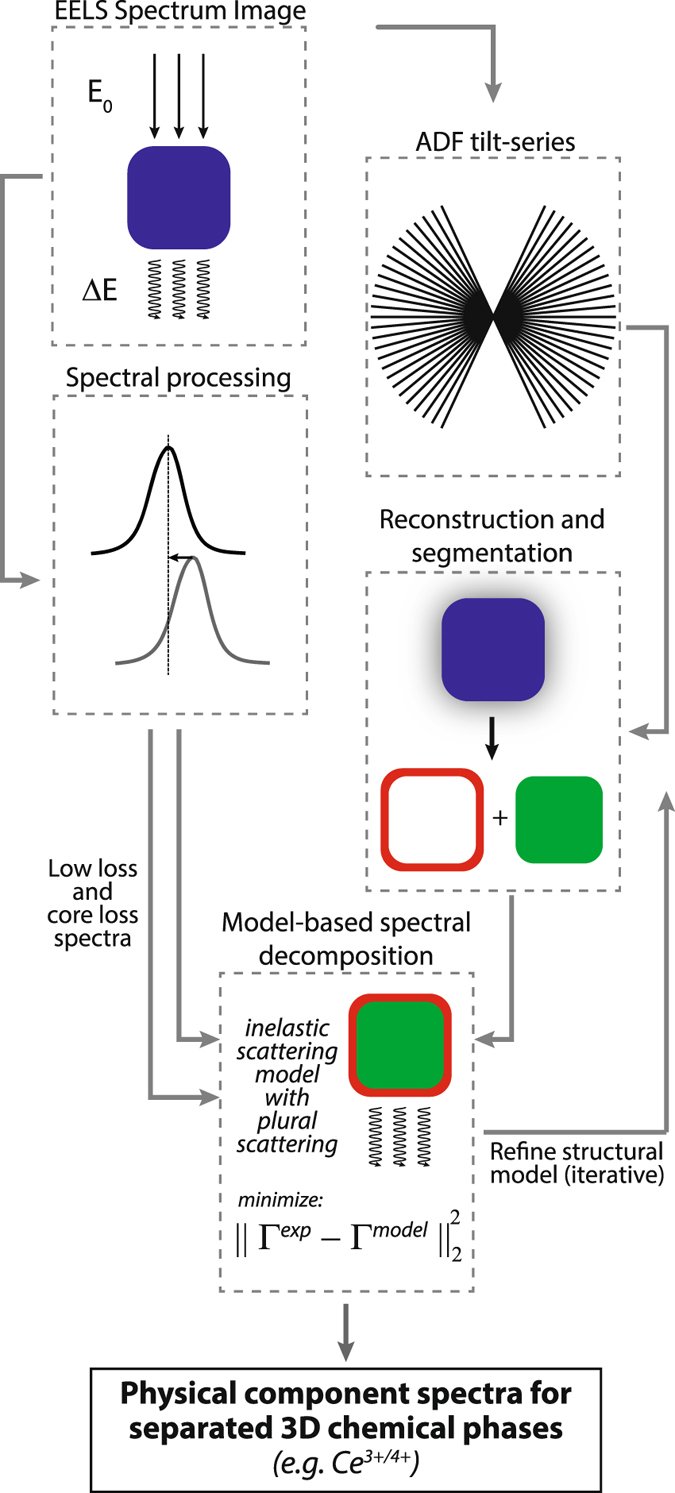
Schematic illustration of a model-based approach to spectroscopic tomography. EELS (left) and ADF-STEM (right) processing are combined to recover spectra, with sub-nanometer precision and comprehensive modeling of plural inelastic electron scattering, corresponding to 3D chemical phases.

In order to make use of such noisy 2D data for 3D microanalysis, a multi-modal protocol was designed to incorporate measurements of the particle morphology. Figure 2 outlines the key processing steps: The EELS spectrum-image was acquired first, and subsequently an ADF-STEM tomographic tilt-series was recorded. Further processing was conducted in parallel to align data and reconstruct the morphology of the particle. The reconstructed morphology was then re-projected to obtain segmented thickness maps for the sample orientation in the recorded spectrum-image. Finally, these multiple elements were combined to optimize a spectral decomposition of the core and shell contributions to the recorded 2D spectrum-image using a model incorporating plural scattering effects, the measured morphology of the sample, and a refined segmentation of the core and shell volume fractions (see also Methods and Supplementary Note 1).

**Figure 3:**
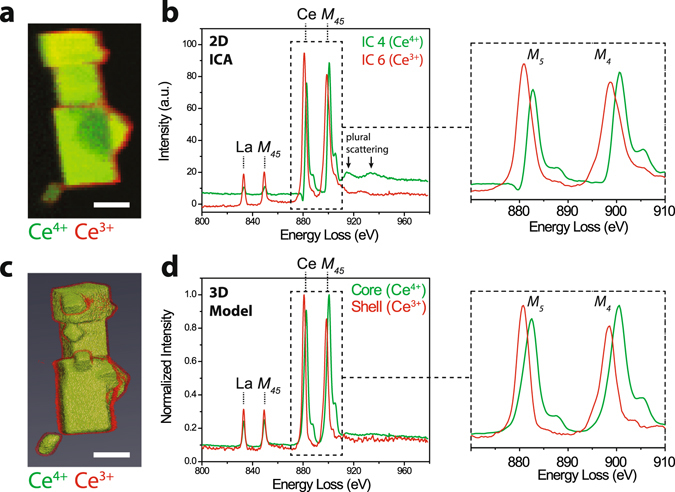
Analysis of composition and oxidation state. (**a**–**b**) Independent component analysis (ICA) of 2D EELS mapping of a La-doped ceria nanoparticle aggregate. The spatial distributions in (**a**) correspond to spectral components in (**b**). Ce *M*_45_ spectral shifts and changes in the *M*_5_/*M*_4_ intensity ratios, signatures of the Ce oxidation state, are highlighted on the right. Residual plural scattering features are marked with arrows. (**c**) 3D volume visualization of reconstructed nanoparticle aggregate refined by a model-based EELS approach. (**d**) The corresponding spectra recovered by model-based EELS tomography. Ce *M*_45_ spectral shifts and changes in the *M*_5_/*M*_4_ intensity ratios, signatures of the Ce oxidation state, are highlighted on the right. Scale bars are 25 nm.

Figure 3 depicts further spectral processing of the data set presented in Fig. 1. In this multi-particle aggregate, the primary particle was oriented with the [100] direction approximately parallel to the electron beam. Here, two analytical approaches are highlighted. First, a 2D multivariate statistical method, independent component analysis (ICA)^24^, was used to analyze the spectrum-image. Two components were identified that corresponded to signatures for Ce^4+^ and Ce^3+^, based on the energies and relative intensities of the Ce *M*_45_ peaks. Figure 3a shows the spatial distribution of the two spectral components presented in Fig. 3b. While useful for extracting these chemical signatures with significantly reduced noise, ICA analysis contained several non-physical features such as irregular dips at energies with significant peak overlap. Moreover, the ICA lacked a clear physical basis for the spectral decomposition. Further, the recovered ICA spectra showed substantial residual contributions from multiple inelastic scattering, precluding the use of the spectra for quantitative analysis. The ICA results did highlight, however, a core-shell spatial separation of the two Ce oxidation states in the sample.

Moving beyond the limited ICA analysis, the model-based spectral decomposition approach outlined in Fig. 2 yielded a 3D spatially resolved chemical separation (Fig. 3c,d). Figure 3c presents a volume visualization of the core and shell chemical phases (see also Supplementary Fig. S1). The spectra recovered in Fig. 3d showed the corresponding physically-based single scattering distribution spectra. In contrast to the ICA analysis, physically meaningful peak shapes were recovered. As in the ICA, signatures of Ce^3+^ were observed in the shell, and signatures of Ce^4+^ in the core. La signals were observed in both the core and in the shell. Notably, the Ce post-edge energy window showed substantially reduced plural scattering effects in the core spectrum. The *M*_5_/*M*_4_ ratios were 1.14 and 0.89 for the shell and core, respectively, consistent with the Ce^3+^ and Ce^4+^ limiting cases for the Ce cation oxidation state^20, 22^. The *M*_45_ line ratios indicated clear phase segregation in the core-shell analysis, with an optimum shell thickness of 1.3 nm.

**Figure 4:**
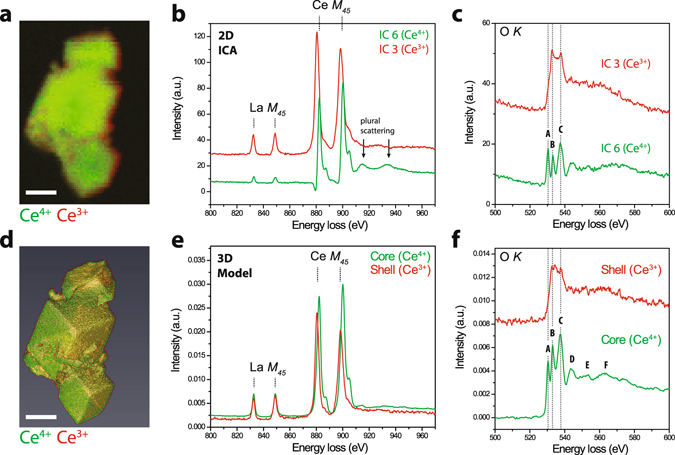
Analysis of orbital hybridization. (**a**–**c**) ICA decomposition for a second La-doped ceria nanoparticle aggregate. (**a**) Spatial distribution maps, (**b**) component spectra at the La and Ce *M*_45_ edges, and (**c**) component spectra at the O *K* edge. Arrows mark plural scattering features. (**d**–**f**) Model-based tomographic EELS analysis: (**d**) 3D volume visualization of the core-shell aggregate, (**e**) corresponding spectra at the La and Ce *M*_45_ edges, and (**f**) corresponding spectra at the O *K* edge (Ce and O edges are both from the second aggregate). Dashed lines and letters indicate spectral features attributed to characteristic orbital hybridization signatures. The three prominent peaks were assigned as (A) Ce(*4f*)-O(*2p*), (B) Ce(*5d e*_*g*_)-O(*2p*), and (C) Ce(*5d t*_2*g*_)-O(*2p*). Scale bars are 25 nm.

The EELS fine structure analysis was extended to examine also the O *K* ionization edge. Figure 4 presents Ce *M*_45_ and O *K* fine structure analyses for a second multi-particle aggregate. As for the first aggregate, Fig. 4a–c shows ICA analyses at these energies, replicating the Ce *M*_45_ analysis for this particle ensemble and illustrating the corresponding ICA decomposition of the O *K* edge spectra. Figure 4c shows spectra for the Ce^3+^ and Ce^4+^ ICA components. The Ce^4+^ component exhibited peak positions, though not relative intensities, consistent with CeO_2_. The peaks labeled A-C arise from Ce(*4f*)-O(*2p*) (A) and Ce(*5d*)-O(*2p*) (B-C) orbital hybridization^25^. Additional peaks were faintly observed in the ICA analysis, also attributed to characteristic Ce-O hybridization^13^. These features were reproduced more clearly in the 3D model analysis (Fig. 4d–f). For this aggregate, the primary particles were oriented with the [111] direction approximately parallel to the electron beam, resulting in an apparently thinner shell in the projection of the 3D visualization (Fig. 4d, see also Supplementary Fig. S1). The *M*_5_/*M*_4_ ratios were 1.19 and 0.90 for the Ce^3+^ and Ce^4+^ model spectra, respectively. In this case, the model-based tomography approach revealed the Ce^3+^ shell with a thickness of 1.5 nm showing simultaneous changes in Ce(*f*) hybridization at the O *K* edge (Fig. 4f). Critically, peak A disappeared in the shell spectrum, suggesting a loss of Ce(*4f*)-O(*2p*) orbital hybridization in the Ce^3+^ surface layer. Additionally, the shell spectrum (Fig. 4f) is consistent with the appearance of additional electronic states at energies between the B and C peaks in Ce^3+^ oxides (see also Supplementary Fig. S2)^25^. For the particle core, Fig. 4f shows well-resolved peaks A-F, also showing an intensity distribution consistent with that of CeO_2_^13, 22^. Supplementary Figure S3 shows similar results at the O *K* edge for the first particle aggregate. However, the particle aggregate in Fig. 4 yielded recovered model spectra with lower noise due to the higher number of pixels available for the reconstruction procedure.

Analysis of the La content was performed using the model-based tomography spectra with plural inelastic scattering effects removed, revealing an enrichment of La at the surface for both particle aggregates (Supplementary Note 3 and Supplementary Fig. S4). For this analysis, the near edge peak structures were avoided due to the confounding effects of the changes in the bonding characteristics. Instead, post-edge intensities were compared where EELS signals are proportional to molar concentration (related by element- and ionisation-specific scattering cross-sections^23^), with La/Ce intensity ratios of 0.25 and 0.29 for the core and shell phases, respectively, for the first particle aggregate and 0.14 and 0.25 for the core and shell phases for the second particle aggregate (see also Supplementary Fig. S3). In terms of molar fractions, the intensity ratios were consistent with ca. 10% La content in the particle aggregates^19^; the intensities, moreover, were more sensitive to subtle La surface enrichment than could be reliably captured by applying inelastic scattering cross-sections (for additional discussion on the limitations on quantification see also Supplementary Note 3). These analyses indicated surface segregation of La in these particle aggregates concomitant with the observed changes to the Ce oxidation state and Ce-O orbital hybridization. Together, these results show that oxygen vacancies occur in LDC in addition to those resulting from La^3+^ doping. These additional oxygen vacancies behave in a similar way to oxygen vacancies in pure CeO_2_ and in highly-doped ceria in terms of the spectroscopically recorded changes in the local electronic structure and in terms of their location at the LDC surface. Despite the low-dopant concentrations examined here, surface segregation of the La cation provides strong evidence for an active role of La cations in determining the reactivity of the surface toward oxygen mobility and depletion under reducing conditions.

## Discussion

The presented analysis extracts detailed spectroscopic information without any assumptions about the chemical signatures arising from the sample coupled with sub-nanometer precision for the determination of the surface layer thickness in LDC nanoparticle aggregates. This method is constrained to systems with relatively well-characterized structural features, such as the surface layer chemistry in nano-ceria, and this approach will likely generalize to other systems where the surface chemistry is of primary interest. The approach is also sufficiently flexible that other imaging modalities such as EELS signatures of volume plasmons might be used for spectroscopic tomography reconstructions of distinct chemical phases in tandem with limited core ionization spectrum imaging. The approach imposes sharp boundaries between the chemical phases analyzed here, a necessary constraint due to the single sample orientation used for spectroscopic analysis. Additional phantom calculations showed successful recovery of shell thickness even without sharp boundaries (see Supplementary Note 6 and Figures S10-S11). Moreover, there is a one-to-one correspondence between the average spectral signatures of the two structurally segmented phases and the recovered spectra. There is a general trade-off between the knowledge of the 3D structure of a multi-phase system and the free parameters allowed during optimization. In this case, the structure was fixed to enable free optimization of the spectral signals and to eliminate the effects of repeated electron exposures. More conventional tilt-series spectral tomographic approaches^16, 17^ maintain greater freedom in the refinement of the morphological reconstruction but require significantly more input data and considerably higher electron doses.

Such flexibility in the reconstruction is important for non-homogeneous dopant profiles as in Fe-doped ceria, examined in ~20 nm particles^17^. In the case of LDC, the dopant distribution is relatively homogeneous, and sensitivity to the changes in spectra of separate chemical phase regions of LDC particles was prioritized. This work on LDC stands out from previous spectral tomography of valence states in <10 nm undoped ceria particles^16^ in that, while showing similar measurements of the thickness of the Ce^3+^ surface layer in ceria, we analyze total volumes an order of magnitude larger in size. Moreover, larger aggregates of nano-scale particles may be possible using this approach by further increasing the incident electron energy to 200–300 kV, enabling the analysis of thicker samples with comparable multiple inelastic scattering properties.

Previous microanalysis of lanthanide-doped ceria has comprised NEXAFS, XPS, and transmission EELS^10–15^. These measurements do not distinguish the 3D chemistry of samples analyzed, and in most cases XPS analyses reflect the chemistry only near the surface. The addition now of model-based EELS analyses of multi-particle aggregates of LDC particles demonstrates that surface characterization of the first few unit cells does not describe the bulk composition or oxidation states in aliovalent doped ceria samples. Conversely, transmission measurements likely miss the fine scale, and spectrally-overlapping, changes in the O *K* edge signatures in these systems, particularly in regions of samples away from particle edges. Furthermore, the bulk O *K* electronic structure recovered here underscores that Ce(*4f*)-O(*2p*) orbital hybridization occurs in the bulk of LDC, a significant observation for density functional theory treatment of doped ceria^26, 27^. Additional work will be required to extend the observations on LDC with low dopant concentrations to other aliovalent or isovalent dopants.

Comparisons between NEXAFS, *in situ* XPS measurements, and *ex situ* EELS indicate that the surface composition and oxidation states vary depending on the chemical environment and pressure at the sample in doped ceria^14^, suggesting dopants may reduce energy barriers to redox processes without directly inducing additional oxygen vacancies beyond those balancing La^3+^ cations. Our 3D model-based results are consistent with a limited role of the La cations and their associated extrinsic oxygen vacancies in the bulk material where the experimental electronic structure resembles that of CeO_2_. Our findings of La surface enrichment, however, reveal additional complexity in that the inhomogeneity of La cations modifies the energetics of redox processes specifically in the first few unit cells at the catalytic surface. The La^3+^ surface enrichment is not plausibly due to vacuum- or electron beam-induced reduction, and therefore represents an additional source of oxygen vacancies at the surface (one oxygen vacancy for every two La^3+^ ions). The relative composition and chemistry of the surface and the bulk is crucial in catalysis applications. Future catalyst designs may target reduced total dopant concentrations in favor of surface enrichment to promote oxygen vacancy formation at reactive surface sites.

The electronic structure and bonding characteristics of multi-particle LDC catalyst aggregates were revealed in 3D with sub-nanometer precision using a model-based spectroscopic tomography strategy. These analyses revealed additional oxygen vacancies within the first 1.5 nm of the catalyst particle surfaces with associated changes in Ce(*4f*) hybridization as well as surface enrichment of La in low-dopant concentration LDC. Cumulatively, we show that La plays a surface-segregated role in the active redox layer of LDC catalyst particles suggesting new design strategies for high efficiency, economically doped ceria-based technologies.

## Methods

### Sample preparation

LDC nanoparticle samples were prepared as reported previously^19^. For electron microscopy, powders were sonicated in diethyl ether and drop-cast onto lacey carbon TEM grids. The grids were dried in air and stored in a vacuum dessicator.

### Data acquisition

STEM-EELS data were acquired using an FEI Osiris equipped with an X-FEG electron source and operated at 80 kV. The beam convergence was 11.0 mrad. EELS was acquired on a Gatan Enfinium spectrometer with a dispersion of 0.25 eV per channel. A 2.5 mm entrance aperture was selected, defining a collection semiangle of 17.9 mrad. Spectra were acquired in dualEELS mode with an exposure time of 0.2 s for core ionization signals and 0.0001 s for the zero loss peak and plasmon loss energies. For ADF tilt-series data, images were acquired every 3° from 70° to −52° for the particle aggregate in Figs 1 and 3 and from 69° to −72° for the particle aggregate in Fig. 4.

### Spectral Processing

Spectral processing was performed using HYPERSPY^28^, an open-source software coded in Python. The spectra were aligned to the zero loss peak (ZLP), first by shifting the maximum intensity channel to zero followed by a cross-correlation based sub-pixel alignment procedure. Spikes due to X-rays striking the charge-coupled device detector were removed using a routine that automatically identified outlying high-intensity pixels and performed interpolation in the spectral region after removal of the spike. Independent component analysis (ICA) was likewise performed in HYPERSPY (see also Supplementary Note 4).

### ADF tomography

ADF tilt-series micrographs were initially aligned using cross-correlation and the tilt-axis was aligned in SCIKIT-IMAGE, an open-source image processing software coded in Python. A compressed sensing reconstruction algorithm coded in MATLAB (Mathworks)^29^ was then used to obtain a reconstruction volume. In brief, the compressed sensing electron tomography algorithm uses prior knowledge of the object sparsity and the sparsity of the object in the gradient transform domain in combination with an under-sampled tilt-series in the Fourier transform domain to recover a high-fidelity tomographic reconstruction from limited projection data. The algorithm is implemented as an ${\ell }_{1}$- and total variation (TV)-regularized Fourier-domain tomographic reconstruction^29, 30^.

The volume was segmented by threshold binarization in ImageJ followed by hole-filling operations using SCIKIT-IMAGE. The binary volume was segmented to give core and shell sub-volumes by applying a gradient kernel in SCIKIT-IMAGE, and the shell thickness was varied by changing the kernel size (see also Supplementary Note 5 and Supplementary Fig. S9). The segmented volumes were re-projected using a numerical Radon transform in SCIKIT-IMAGE and registered with the experimental spectrum-image data using routines in MATLAB.

### Model-based tomography

The total electron scattering contributions as a function of energy loss *E* from electrons scattered *n* times can be written generally as a sum of *n*-fold scattering distributions (see also Supplementary Note 1)^23, 31, 32^.

For a two-phase sample, the expression is appropriately further expanded to account for the distinct single scattering contributions *p*_1*a*_(*E*) and *p*_1*b*_(*E*):

1 $${\rm{\Gamma }}(E)={K}_{0}\,(\frac{{t}_{a}}{{\lambda }_{a}})\,{p}_{1a}(E)+{K}_{0}\,(\frac{{t}_{b}}{{\lambda }_{b}})\,{p}_{1b}(E)+{{\bf{H}}}_{{\rm{LL}}}\,[(\frac{{t}_{a}}{{\lambda }_{a}})\,{p}_{1a}(E)+(\frac{{t}_{b}}{{\lambda }_{b}})\,{p}_{1b}(E)],$$

where *K*_0_ is the intensity of electrons undergoing no inelastic scattering, *t*_*a*_ and *t*_*b*_ are the thicknesses and *λ*_*a*_ and *λ*_*b*_ are the inelastic mean free paths of materials *a* and *b*, respectively, and **H**_LL_ is a matrix describing the convolution of core loss ionization scattering distributions with the distribution of inelastic scattering at low energy losses.

Given experimental data at many pixels *i* with both low loss and high loss spectra recorded for each pixel, the single scattering distributions can be recovered by solving the minimization problem:

2 $$[{p}_{1a},{p}_{1b}]={\rm{\arg }}\,{{\rm{\min }}}_{[{p}_{1a},{p}_{1b}]}\{{\Vert {{\rm{\Gamma }}}_{i}^{\exp }-{{\rm{\Gamma }}}_{i}^{{\rm{calc}}}\Vert }_{2}^{2}\}\mathrm{.}$$

Without considering multiple scattering effects, and retaining only the first two terms in equation (1), the problem is overdetermined where the number of pixels *i* exceeds the number of phases, assuming independent measurements at different pixels. With plural scattering effects, the problem is no longer linear and the recovery at each energy channel is no longer independent due to the convolution terms, further constraining the problem. In any case, the problem will be highly overdetermined for typical spectrum images with hundreds or thousands of pixels recorded.

This spectral recovery problem was implemented using a nonlinear conjugate gradient algorithm in SCIPY, an open-source software coded in Python. The analytical gradient of equation (2) was determined for efficient computations. Spectral recovery was terminated when machine precision was lost, typically after 20–40 iterations, sufficient for high quality spectral information recovery from noisy input data. For noise-free and low-noise phantom data (not shown), iterations were continued until a gradient tolerance (e.g. 1 × 10^−5^) was obtained. Phantom data sets were used to validate equation (1) for use in the recovery problem in equation (2). These phantom data sets consisted of plural scattering spectra constructed first by summation of *n*-fold scattering distributions up to four-fold scattering followed by recovery using the convolution matrix formulation. For noise-free phantom data, the recovery enabled correct spectral retrieval to within machine precision.

Thickness information was obtained from segmented structural electron tomograms (ADF tomography), and mean free paths were fixed at estimated values determined by examining the zero loss and plasmon loss spectra to assess the *t*/*λ* ratio. This segmentation procedure imposed sharp boundaries in the reconstruction, suggesting an abrupt interface between the core and shell chemical domains. Additional phantom calculations were carried out to validate the method (see Supplementary Note 6 and Figures S10-S11). These calculations indicated valid recovery even for a gradient in chemical composition at the boundary.

Matrix formulations of the convolution matrix for the recovery problem resulted in large memory requirements for 2000–5000 pixels and the large energy windows used in the spectral recovery problem (up to approximately 400 eV at 0.25 eV/channel dispersion). To reduce the memory requirements, zero-thickness pixels were excluded from processing. The convolution matrix was also truncated to the square size of the high loss energy window of interest. For the spectrum-image sizes and energy ranges studied, the memory required in the present implementation was approximately 30–50 Gb.

Due to the formulation in terms of inelastic scattering events, the zero loss peak was removed by imposing a low energy cut-off in the plasmon energy range at 5.0 eV. For O *K* edge analysis, the pre-edge scattering intensity was also background subtracted at energies below the ionization edge by power law fitting.

### Data availability

The datasets generated during and/or analysed during the current study are available from the corresponding author on reasonable request.

## Electronic supplementary material

Supplementary Information
