# Supplementary material for: Sub-nanometer surface chemistry and orbital hybridization in lanthanum-doped ceria nano-catalysts revealed by 3D electron microscopy
Source: Sci Rep. 2017 Jul 14;7:5406. doi: 10.1038/s41598-017-05671-9 (PMC5511253; doi:10.1038/s41598-017-05671-9)
Supplement: Supplementary file 1 — Supplementary Information [file 41598_2017_5671_MOESM1_ESM.pdf]

Supplementary Information for "Sub-nanometer surface chemistry and orbital hybridization in lanthanum-doped ceria nano-catalysts revealed by 3D electron microscopy"

Sean M. Collins,[1,*] Susana Fernandez-Garcia,[2]
José J. Calvino[2] and Paul A. Midgley[1,*]

[1] *Department of Materials Science and Metallurgy, University of Cambridge, 27 Charles Babbage Road, Cambridge, CB3 0FS, United Kingdom*

[2] *Departamento de Ciencia de los Materiales, Ingeniería Metalúrgica y Química Inorgánica, Facultad de Ciencias, Universidad de Cadiz, Campus Río San Pedro, Puerto Real (Cádiz) E-11510, Spain*

* Email: smc204@cam.ac.uk, pam33@cam.ac.uk

Contents

1 Inelastic electron scattering model for a two-phase sample 2

2 Supplementary Figures 4

3 La Surface Enrichment 6

4 Independent Component Analysis (ICA) 7

5 Model Optimization 10

6 Phantom Calculations 10

1 Inelastic electron scattering model for a two-phase sample

The total electron scattering contributions as a function of energy losses E from electrons scattered n times can be written generally as:[1–5]

$$\Gamma(E) = \sum_{n=0}^{\infty} K_n p_n(E), \tag{S1}$$

where K_n gives the coefficient weighting the n-fold scattering distribution $p_n(E)$.

The coefficient is given by Poisson statistics, such that:[5,6]

$$K_n = \frac{1}{n!}\left(\frac{t}{\lambda}\right)^n \exp\left(-t/\lambda\right), \tag{S2}$$

where t is the thickness and λ is the inelastic mean free path of the electron.

As such, the first two coefficients are given as:

$$\begin{aligned} K_0 &= \exp\left(-t/\lambda\right) \\ K_1 &= \left(\frac{t}{\lambda}\right)\exp\left(-t/\lambda\right) = \left(\frac{t}{\lambda}\right)K_0. \end{aligned} \tag{S3}$$

The n-fold scattering distributions are given by the recurrence relation:

$$p_{n+1}(E) = p_n(E) * p_1(E), \tag{S4}$$

giving the plural scattering terms as n-fold convolutions of the single scattering distribution $p_1(E)$.

The sum over inelastic scattering contributions to the total scattering can be expanded as:

$$\begin{aligned} \Gamma(E) = K_0\left(\frac{t}{\lambda}\right)p_1(E) + K_0\left(\frac{t}{\lambda}\right)^2\left(\frac{1}{2}\right)p_1(E) * p_1(E) \\ +K_0\left(\frac{t}{\lambda}\right)^3\left(\frac{1}{6}\right)p_1(E) * p_1(E) * p_1(E) + \ldots. \end{aligned} \tag{S5}$$

In EELS, the experimental spectrum at low energy losses records the plural scattering contributions to convolutions with electrons scattered to high energy losses (HL), typically core ionisation events. For HL inelastic scattering, the expression can be simplified as a single scattering distribution and a convolution with the low loss (LL) spectrum. With the convolution matrix written as $\mathbf{H}_{\mathrm{LL}}$, the HL scattering distribution is then given as:

$$\Gamma_{\mathrm{HL}}(E) = K_0\left(\frac{t}{\lambda}\right)p_1(E) + \mathbf{H}_{\mathrm{LL}}\left(\frac{t}{\lambda}\right)p_1(E). \tag{S6}$$

Whereas previous treatments of the convolution and deconvolution process have handled the sample as a single phase material,[1–6] a successful approach for two-dimensional

electron microscopy, the description requires a modification for separately handling two phases for use in combination with three-dimensional segmented volumes in a spectroscopic tomography method. For a two-phase sample, the expression is appropriately further expanded to account for the distinct single scattering contributions $p_{1a}(E)$ and $p_{1b}(E)$:

$$\begin{aligned}\Gamma(E) = K_0 \left(\frac{t_a}{\lambda_a}\right) p_{1a}(E) + K_0 \left(\frac{t_b}{\lambda_b}\right) p_{1b}(E) \\ +\mathbf{H}_{\mathrm{LL}} \left[\left(\frac{t_a}{\lambda_a}\right) p_{1a}(E) + \left(\frac{t_b}{\lambda_b}\right) p_{1b}(E)\right].\end{aligned} \tag{S7}$$

2 Supplementary Figures

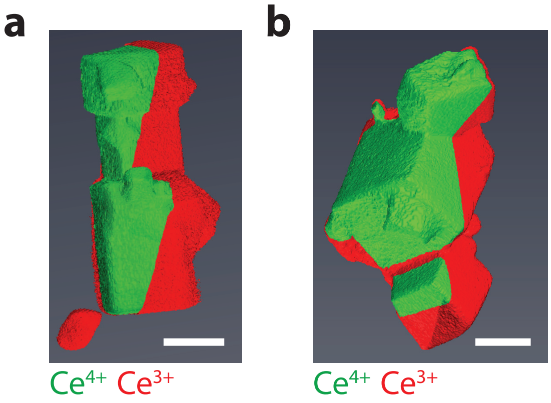

Figure S1 Alternative 3D visualization of the optimized core/shell structures in La-doped ceria nano-catalysts. The segmented shell volume has been cropped at an oblique plane. (a) Aggregate in Figure 3. (b) Aggregate in Figure 4. Scale bars are 25 nm.

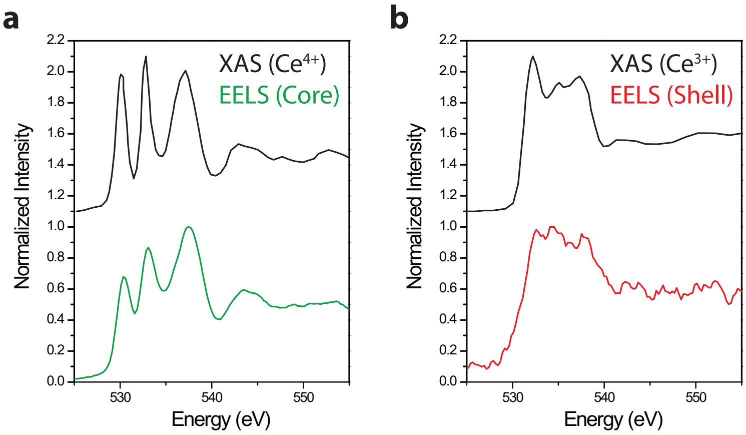

Figure S2 Comparison of X-ray absorption spectroscopy (XAS) and EELS of Ce^{4+} and Ce^{3+} oxides. XAS (black) and EELS determined from model-based tomography corresponding to the (a) core (green) and (b) shell (red) as in Figure 3. XAS data reproduced from Ref. [7].

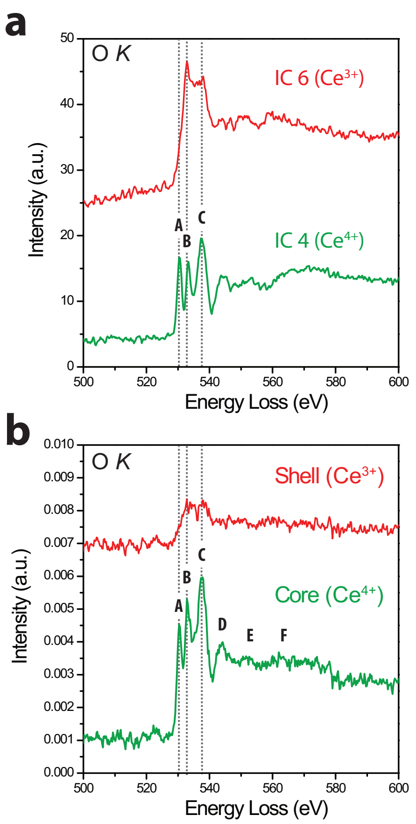

Figure S3 O *K* edge analysis for the La-doped ceria nanoparticle aggregate in Figure 1 and Figure 3 (first aggregate). (a) ICA spectra and (b) model-based spectra. The signal-to-noise ratio was lower in this data-set than for the aggregate in Figure 4 (second aggregate) because fewer pixels were available for use in the model-based optimization.

3 La Surface Enrichment

EELS signals are conventionally quantified by applying element- and ionisation edge-specific inelastic scattering cross-sections.[5] Such cross-sections may be calculated at different levels of theory, with hydrogenic and Hartree-Slater cross-sections among the most common approaches.[5] Hartree-Slater cross-sections indicated the observed intensities were consistent with ca. 10% La content. The variations in the observed intensities (see Figure S4) showed fine-scale surface enrichment of La. This variation was on a finer scale than acceptable for reliable application of the Hartree-Slater cross-sections, due to several significant sources of error in treating these ionisation edges. The edges for La and Ce overlap in energy, requiring separation of the lower-energy La ionisation edges from the Ce intensities with only a narrow energy window between the strong peaks at the M_{45} edges which are simultaneously highly dependent on the oxidation state. Moreover, the strong peaks are not captured in Hartree-Slater cross-sections. These issues make the application of ionisation cross-sections an overly simplistic and limited approach for quantification of the intensities. Direct comparison of the intensities in Figure S4 represents the compositional variations without bias from any inaccuracies in modelling the scattering distribution.

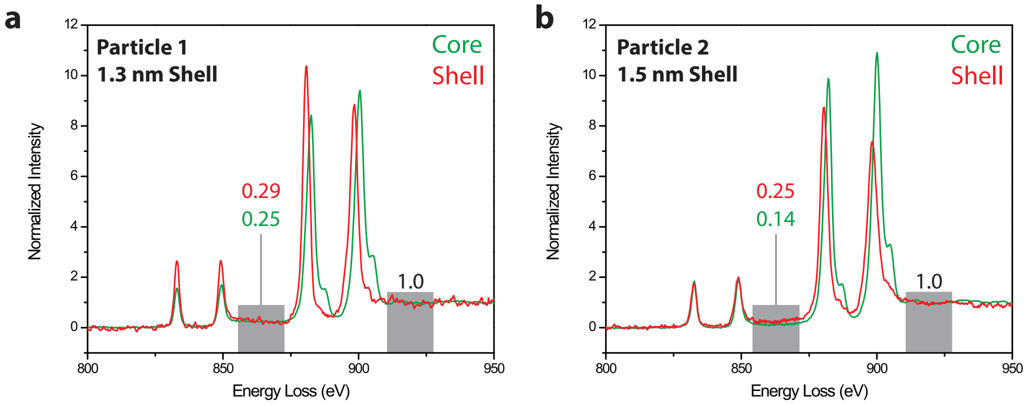

Figure S4 Normalized La post-edge intensities. Pre-edge background subtracted for (a) Particle 1 (Figure 1, Figure 3) and (b) Particle 2 (Figure 4). The grey boxes indicate the post-edge spectral windows used to examine the La/Ce intensities. The intensities in the Ce post-edge window were normalized to 1.0.

4 Independent Component Analysis (ICA)

ICA consists of a two-step procedure. First, principal component analysis (PCA) is performed in order to identify components according to the variance properties in the data set to yield a linear model of the data. Subsequently, ICA algorithms retrieve mutually independent components.[8] In PCA, a plot of the component eigenvalues, termed a 'Scree plot,' can be used to assess the number of components needed to describe the data set. Large values are expected to stand out above a background of noise-related components. By analysing the Scree plot, the number of components for ICA can then be selected systematically.

In many cases, STEM-EELS spectrum-images are suitable for ICA.[8] Plural scattering effects, however, are not readily handled by ICA algorithms. While, in principle, the contributions from plural inelastic electron scattering are additive, the spectra depend singificantly on the thickness of the sample when plural scattering contributions are non-negligible. Unless the sample consists of only a few thicknesses, the ICA algorithm cannot decompose the spectra into only a few components. Here, the samples exhibited a continuous range of thicknesses through the spectrum images, resulting in the residual plural scattering contributions in Figure 3-4 in addition to the ICs which separate plural scattering effects from regions of similar thickness (Figure S6, Figure S8).

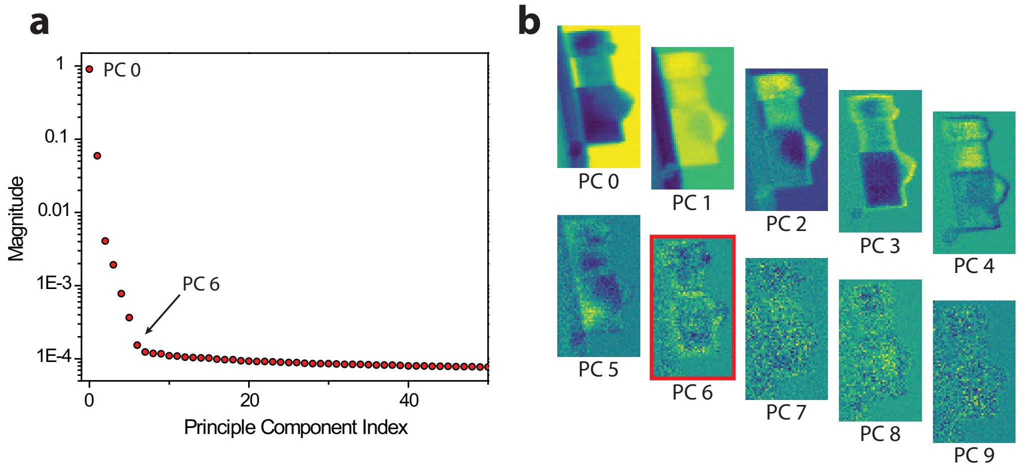

Figure S5 Principal component analysis of Particle 1. (a) Scree plot and (b) corresponding spatial distribution maps for the first 10 principal components (PCs). PC6 was identified as the last significant component.

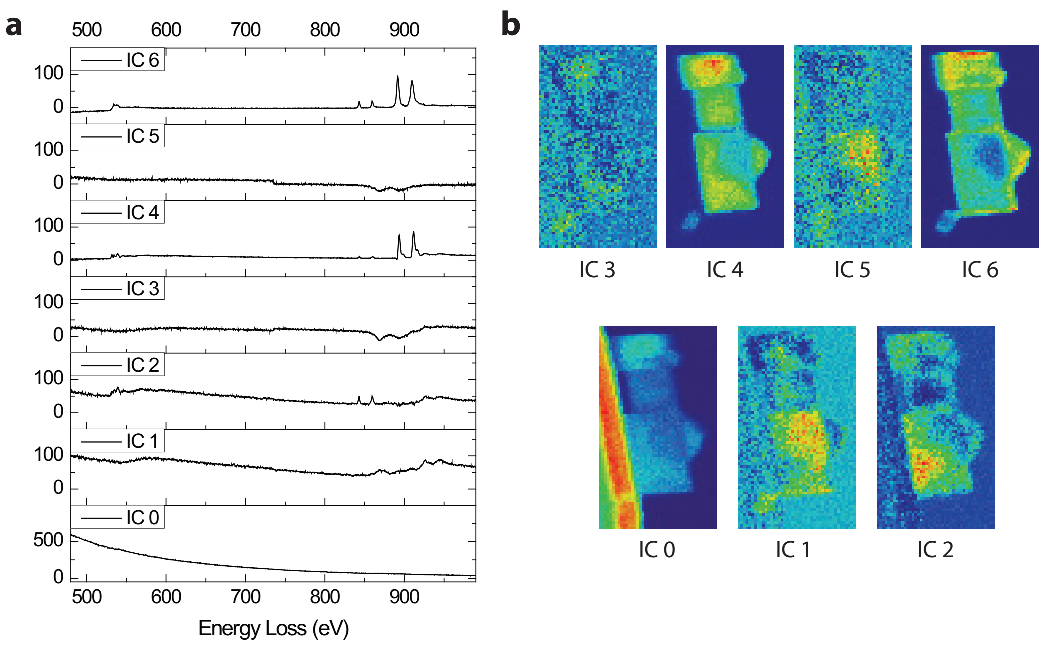

Figure S6 ICA results for Particle 1. (a) Indepent component (IC) spectra and (b) corresponding spatial distribution maps for the seven retained ICs.

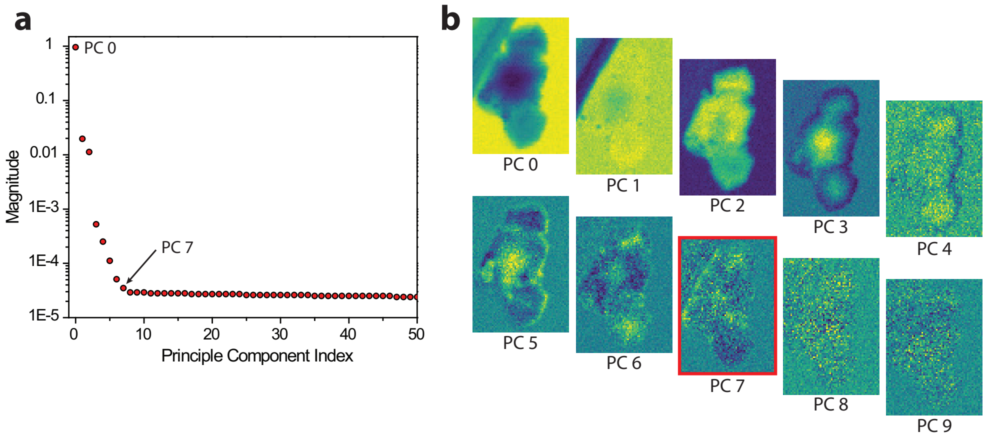

Figure S7 Principal component analysis of Particle 2. (a) Scree plot and (b) corresponding spatial distribution maps for the first 10 PCs. PC7 was identified as the last significant component.

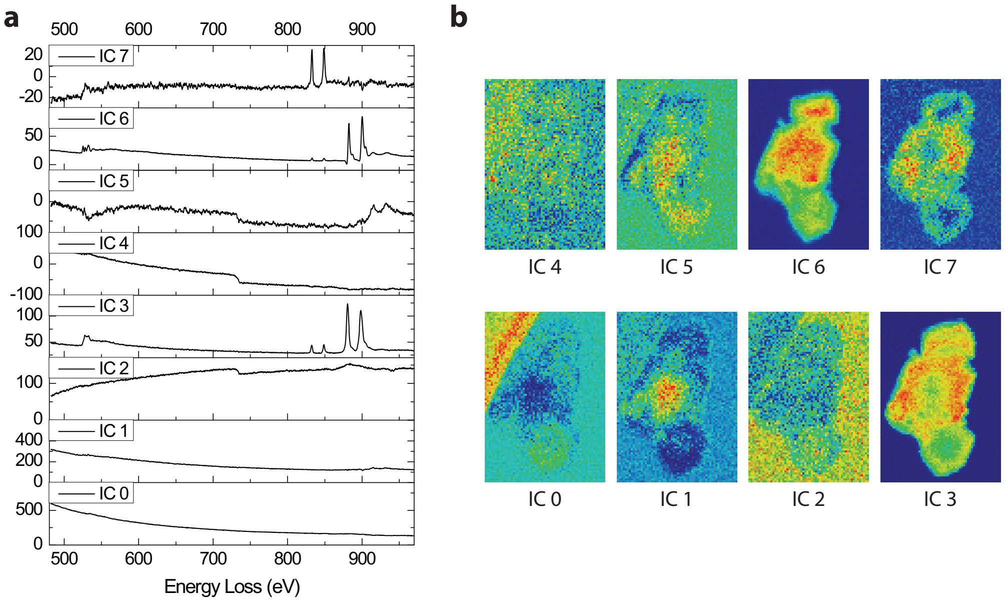

Figure S8 ICA results for Particle 2. (a) IC spectra and (b) corresponding spatial distribution maps for the eight retained ICs.

5 Model Optimization

The structural model was optimized by incrementally adjusting the shell thickness (see Figure 2, Experimental Section). When the model shell was too thin, unphysical asymmetries appeared in the recovered spectra (dashed arrows, Figure S9). When the model shell was too thick, the M_{45} peak energies and intensities shifted toward the Ce^{4+} signature and exhibited the high energy satellite peaks characteristic of the Ce^{4+} state (solid arrows, Figure S9).

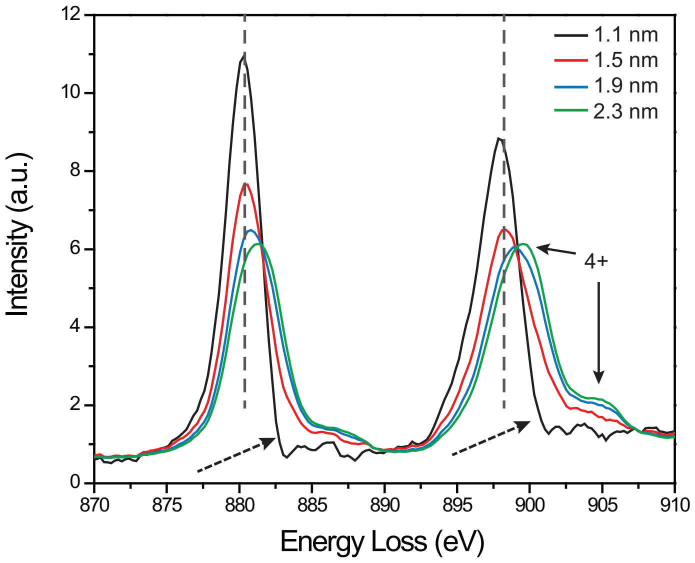

Figure S9 Example model optimization. The shell thickness for Particle 2 was incremented to determine the optimal shell thickness of 1.5 nm. Dashed lines highlight the M_{45} peak energies characteristic of Ce^{3+}. Dashed arrows mark unphysical peak asymmetries. Solid arrows mark Ce^{4+} signatures.

6 Phantom Calculations

In order to further test the validity of the reconstruction method, and to examine particularly the role of the sharp-boundary segmentation imposed in the method, a series of phantom calculations were carried out. Such phantom calculations allow for comparison of a method with a know true solution. In this case, experimentally recovered low-noise spectra at the Ce M_{45} edge were selected as end-member spectra representing Ce^{3+} and Ce^{4+}. In order to investigate specifically the effect of an abrupt or non-abrupt interface on the reconstruction using abrupt (sharp-boundary) segmentations, multiple scattering contributions were not considered in these phantom calculations. Figure S10 presents

the results of phantom calculations, one with an abrupt interface (Fig. S10a) and a second with an interface with linearly varying composition between the core and the shell (Fig. S10b).

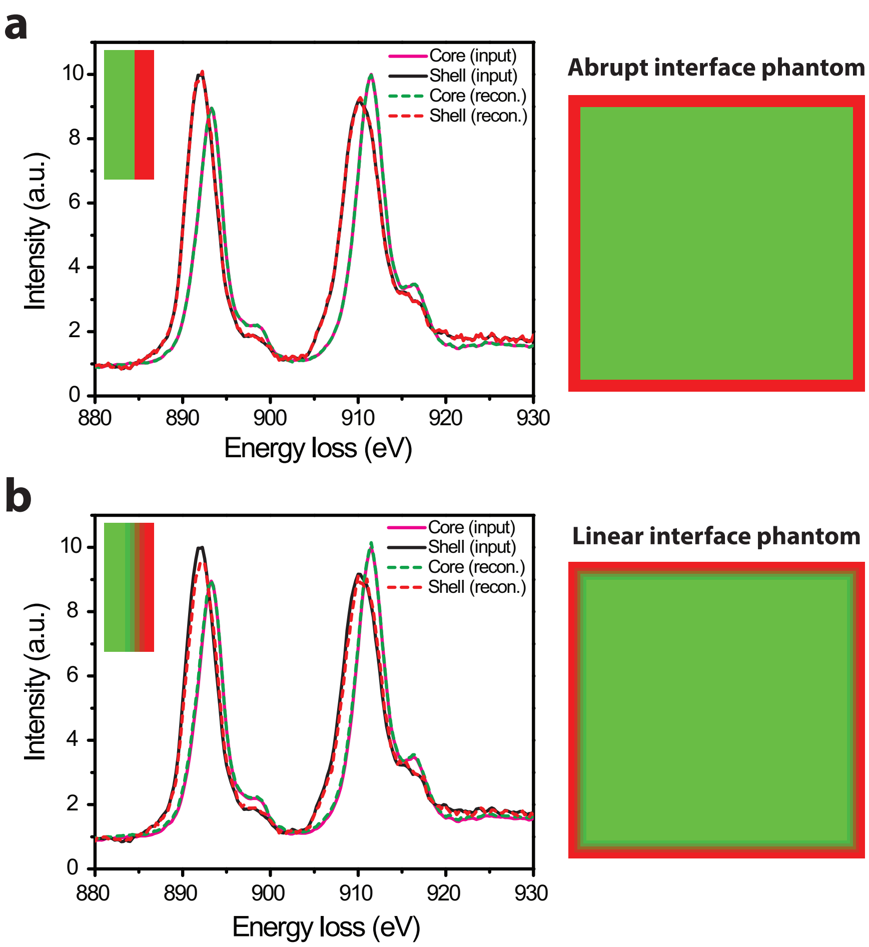

Figure S10 Reconstruction spectra for two-dimensional phantom structures with **a** an abrupt interface and **b** a linear interface region. The insets show the interface, and the entire two-dimensional phantom (one-dimensional projection data). The spectra used in generating the phantom (input) are given for reference.

The phantoms each consisted of a two-dimensional square, the analog of a cubic particle. The presented method does not take into account the dimensionality of the problem (Fig. 2); the input for spectral recovery is a set of spectra from N pixels and the corresponding segmented thickness maps. The phantoms had a side-length of 100 pixels, defined as 50 nm in physical extent in analogy to the experimental nanoparticles. The true shell thickness was fixed at 2 nm. In the linear interface phantom, the interface region was ± 1 nm (2 nm interface region). The end-member spectra were weighted at

each pixel in the phantom according to these parameters. To simulate experimental conditions, the phantom spectrum images were then projected and Poissonian noise was added to the spectra. The spectra and corresponding thickness profiles were used in the reconstruction algorithm.

Figure S10 shows the recovered spectra. In both cases, the end-member spectra were recovered to high accuracy. For the linear interface phantom, the reconstructed shell spectrum showed a slight deviation in the low energy peak relative to the input shell spectrum. This reduced performance of the reconstruction method for a non-abrupt interface is expected. However, the effect appears relatively minor in the phantom.

To test whether a linear interface enables correct identification of the shell thickness, a further series of simulations were carried out. In this series, the shell thickness was varied using the same linear interface phantom spectrum image data. Figure S11 presents the recovered spectra for the shell as in the optimization of the experimental model in Fig. S9.

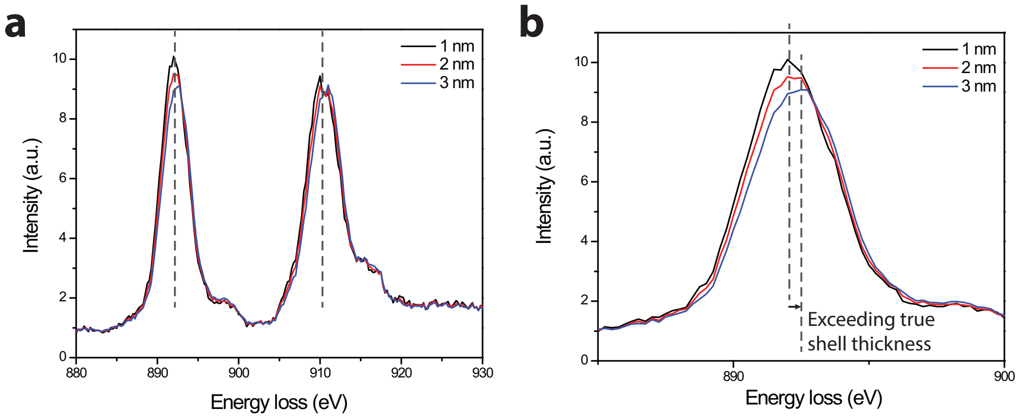

Figure S11 Model optimization for the linear interface phantom. **a**, Reconstructed spectra for shell thickness values in the range 1-3 nm. **b**, An enlarged view of the low-energy peak, highlighting a significant shift in the peak energy at 3 nm shell thickness (exceeding the true shell thickness of 2 nm).

The optimization of the shell thickness for the linear interface phantom bears a significant resemblance to the experimental optimization. For shell thickness estimates exceeding the optimal or, in the case of the phantom, the true shell thickness, the peak energies shift substantially. For thicknesses that are thinner, the spectra show some variation in the relative intensities of the peaks. However, the peak energies remain approximately constant up to and including the true thickness for the phantom. Based on these findings on the phantom, the experimental identification of the shell thickness is valid even for a gradual transition from the Ce^{3+} shell to the Ce^{4+} core. Moreover, in the experimental case, the optimized shell thickness corresponded to reported end-member relative peak intensities, suggesting a minimal effect due to any non-abrupt boundary

between the core and shell chemistries.

References

1. Misell, D. L. & Jones, A. F. The determination of the single-scattering line profile from the observed spectrum. *J. Phys. A: Gen. Phys.* **2,** 540 (1969).
2. Schattschneider, P. Retrieval of single-loss profiles from energy-loss spectra A new approach. *Philos. Mag. B* **47,** 555–560 (1983).
3. Schattschneider, P. A performance test of the recovery of single energy loss profiles via matrix analysis. *Ultramicroscopy* **11,** 321–322 (1983).
4. Verbeeck, J. & Bertoni, G. Deconvolution of core electron energy loss spectra. *Ultramicroscopy* **109,** 1343–1352 (2009).
5. Egerton, R. F. *Electron Energy-Loss Spectroscopy in the Electron Microscope* 3rd ed. 2011 (Springer, New York, 2011).
6. Su, D. S. & Schattschneider, P. Deconvolution of angle-resolved electron energy-loss spectra. *Philosophical Magazine A* **65,** 1127–1140 (1992).
7. Mullins, D. R, Overbury, S. H & Huntley, D. R. Electron spectroscopy of single crystal and polycrystalline cerium oxide surfaces. *Surf. Sci.* **409,** 307–319 (1998).
8. De la Peña, F. *et al.* Mapping titanium and tin oxide phases using EELS: An application of independent component analysis. *Ultramicroscopy* **111,** 169–176 (2011).
